# Using structural equation modeling to explore the influences of physical activity, mental health, well-being, and loneliness on Douyin usage at bedtime

**DOI:** 10.3389/fpubh.2023.1306206

**Published:** 2024-01-05

**Authors:** Hongcheng Luo, Xing Zhang, Songpeng Su, Mingyang Zhang, Mingyue Yin, Siyuan Feng, Rui Peng, Hansen Li

**Affiliations:** ^1^School of Physical Education, Xichang University, Xichang, China; ^2^Department of Physical Education and Sport, Faculty of Sport Sciences, University of Granada, Granada, Spain; ^3^School of Athletic Training, Guangzhou Sport University, Guangzhou, China; ^4^Digitalized Strength and Conditioning Training Laboratory, Guangzhou Sport University, Guangzhou, China; ^5^Institute of Sports Science, Jishou University, Jishou, China; ^6^School of Athletic Performance, Shanghai University of Sport, Shanghai, China; ^7^Laboratory of Genetics, University of Wisconsin-Madison, Madison, WI, United States; ^8^McGill University, Montreal, QC, Canada; ^9^Institute of Sports Science, College of Physical Education, Southwest University, Chongqing, China

**Keywords:** physical activity, public health, mental health, social media, domain-specific behaviors

## Abstract

Douyin is the Chinese version of TikTok. Using Douyin at bedtime is a very common behavior among Douyin users. However, the reasons why users like using Douyin before sleep are yet unclear. We conducted a cross-section survey from January 1st to January 16th, 2023 to capture data to examine the associations of depression, anxiety, life satisfaction, well-being, loneliness, and physical activity with Douyin usage at bedtime. The mediation role of insomnia in these associations was examined. A total of 3,392 participants who met the inclusion criteria were included for analysis. Our structural equation modeling analysis showed that depression on (*β* = 0.08; *p* < 0.05), anxiety (*β* = 0.06; *p* < 0.05), and loneliness (*β* = 0.14; *p* < 0.05) were directly associated with increased Douyin usage at bedtime, and were also indirectly associated with Douyin usage through insomnia (depression: *β* = 0.18; *p* < 0.05, anxiety: *β* = 0.16; *p* < 0.05, and loneliness: *β* = 0.12; *p* < 0.05). Life satisfaction (*β* = −0.05; *p* < 0.05) and well-being (*β* = −0.20; *p* < 0.05) were directly associated with decreased Douyin usage at bedtime, and were also indirectly associated with Douyin usage through insomnia (life satisfaction: *β* = −0.09; *p* < 0.05, and well-being: *β* = −0.11; *p* < 0.05). However, physical activity was unexpectedly associated with increased Douyin usage at bedtime (*β* = 0.20; *p* < 0.05). In conclusion, our findings shed new light on the specific reasons why Douyin users like using Douyin at bedtime.

## 1 Introduction

Douyin, the Chinese version of TikTok, is an emerging short-video sharing platform. Currently, this type of short-video sharing platform has gained worldwide popularity. According to data from the China mobile internet big data company, as of May 2023, Douyin boasts over 700 million active users in China, dedicating an average of 36.6 h per month to the platform (1). It has already become the most widely used social media app in China (1). Worldwide, the international version of Douyin (TikTok) is available in over 160 countries, with a user base of 1.677 billion as of 2023 (2). Given such influence, some governments even use it as a platform for policy announcements (3), making it an integral part of daily life. Furthermore, based on our topic search in the Web of Science database, there has been a significant rise in publications related to Douyin/TikTok (4). In 2020, 58 studies were published; this number surged to 230 studies in 2021 and further increased to 459 studies in 2022 (4). Therefore, there is an increasing research interest in Douyin.

In the past few years, numerous studies have been conducted to investigate the impact of Douyin on public health. For instance, Sha and Dong (5) investigated Douyin’s effects on adolescents, revealing that excessive Douyin usage may lead to mental health problems and memory loss. Zhang et al. (6) uncovered that nighttime Douyin use was positively associated with delayed sleep and poor sleep quality. Additionally, other negative consequences arising from Douyin use have come into focus, including anorexia (7), cyberbullying (8), and negative mood (9). These findings consistently highlight the adverse effects of Douyin usage. However, there is less knowledge about the factors driving Douyin usage, particularly domain-specific usage behaviors.

Using Douyin at bedtime is a domain-specific use behavior that is prevalent among Douyin users. A cross-sectional study conducted by Zhang et al. (6) revealed that more than 85% of Douyin users engage in this use behavior. According to previous studies, mental health problems, such as depression and anxiety, are common factors linked to excessive social media use behavior. Regarding Douyin, Zhang et al. (6) also suggested a potential association between mental health and nighttime Douyin usage behavior. Thus, we formulated the hypothesis (H1) that depression and anxiety are associated with increased Douyin usage at bedtime. Well-being and life satisfaction have also been associated with excessive social media use (10). Previous studies have demonstrated that well-being and life satisfaction can be used to predict social media addiction (11, 12). Considering that Douyin dependence at night might be one manifestation of social media addiction, we formulated the hypothesis (H2) that well-being and life satisfaction are negatively linked to Douyin usage at bedtime. Loneliness is another risk factor for excessive social media use, as individuals experiencing loneliness tend to use social media more frequently than their counterparts (13). Currently, there is limited research on the relationship between Douyin usage at bedtime and loneliness. However, some studies suggest that people may use social media at night due to feelings of loneliness resulting from missing out on messages (14, 15). Therefore, we formulated the hypothesis (H3) that there is an association between loneliness and increased Douyin usage at bedtime.

**Figure 1:**
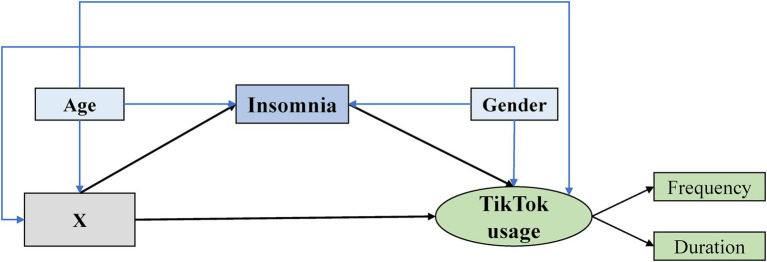
Conceptual framework (Black lines indicate pathways between core variables, and blue lines indicate confounding pathways between core and control variables. X, independent variables such as depression, anxiety, and loneliness.).

Physical activity is typically considered a potential protective factor against excessive social media use (16, 17). However, there are currently fewer researchers focusing on the relationship between physical activity and nighttime Douyin usage. In this aspect, only some researchers suggest that the stress-reducing benefits of physical activity might have a positive impact on alleviating nighttime Douyin usage (6). Therefore, we formulated the hypothesis (H4) that there is a negative association between physical activity and Douyin usage at bedtime. Finally, insomnia may play a crucial mediating role between the aforementioned factors and Douyin usage. This is because insomnia is not only predicted by depression (18, 19), anxiety (18, 19), well-being (20), life satisfaction (21), loneliness (22), and physical activity (23), but is also associated with excessive social media use (24), especially during bedtime (25). Thus, we formulated the hypothesis (H5) that insomnia acts as a mediator between the variables of interest and Douyin usage at bedtime. Given the possibility of confounding in these relationships (26, 27), we included age and gender as covariates. According to our hypotheses, we developed conceptual models as follows (Figure 1).

## 2 Materials and methods

### 2.1 Study design and participants

We conducted a cross-section survey from January 1st to January 16th, 2023. Initially, a preliminary questionnaire was developed by the author(s) following a literature review. Subsequently, this questionnaire was refined based on the feedback received from a pilot study involving 17 participants. The final formal questionnaire was uploaded to the “Sojump” (www.sojump.com). The Sojump is one of the biggest professional online platforms for questionnaire design, questionnaire distribution, data collection, and data analysis in China (28). To distribute the questionnaire, we enlisted the help of 45 college students, who shared a quick response (QR) code linked to our survey through various online chat groups, including WeChat, Tencent QQ, and other social media platforms. We described the study’s topic as investigating reasons why people like using Douyin. Details of the research questions were not disclosed during participant recruitment. We offered compensation of 5 CNY (approximately 0.8 USD) for completing the survey. Participants were required to use WeChat accounts that liked their personal IDs to fill out the questionnaires. Their device, WeChat ID, and IP address were restricted to avoid repeated participation. This study was approved and supervised by the Ethics Review Board of Southwest University.

Inclusion criteria: (i) Age ≥ 18 years; (ii) Douyin usage of at least 1 h per day in the past month. Exclusion criteria: (i) Absence of informed consent; (ii) Unfinished questionnaires; (iii) Individuals who failed verification tests (to confirm that the questionnaire was carefully completed by people instead of a machine or program or random filling).

### 2.2 Instruments and measurements

#### 2.2.1 Depression

In this study, we assessed depression using the Patient Health Questionnaire (PHQ-9) (29). The PHQ-9 comprises nine questions based on the nine criteria for a major depressive episode outlined in the DSM-IV. Each question prompts respondents to indicate the frequency of depressive symptoms they experienced in the 2 weeks preceding the survey, with scores ranging from 0 (not at all) to 3 (nearly every day). We utilized a Chinese version of the PHQ-9, which demonstrated strong internal consistency in the current study (Cronbach’s α > 0.9).

#### 2.2.2 Anxiety

In this study, we assessed anxiety using the Generalized Anxiety Disorder (GAD-7) (30). The GAD-7 is a self-report instrument consisting of seven items. Each item corresponds to one of the hallmark symptoms of generalized anxiety disorder (GAD) and is rated based on the frequency of experiencing that symptom over the preceding 2 weeks (Not at all, 1 = Several days, 2 = More than half the days, and 3 = Nearly every day). We utilized a Chinese version of the GAD-7, which demonstrated strong internal consistency in the current study (Cronbach’s α > 0.9).

#### 2.2.3 Life satisfaction

In this study, we assessed life satisfaction using the Satisfaction with Life Scale-5 (SWLS-5). This scale comprises five self-report items designed to evaluate an individual’s overall satisfaction with their life. Participants were instructed to rate their agreement with each statement on a 7-point Likert scale (1 = Strongly Disagree, 2 = Disagree, 3 = Slightly Disagree, 4 = Neither Agree nor Disagree, 5 = Slightly Agree, 6 = Agree, 7 = Strongly Agree). We utilized a Chinese version of the SWLS-5 which demonstrated strong internal consistency in the current study (Cronbach’s α > 0.9).

#### 2.2.4 Well-being

In this study, we assessed well-being using the World Health Organization-Five Well-Being Index (WHO-5). This index consists of five self-report items designed to measure an individual’s overall sense of well-being and positive mental health. Participants were asked to rate their feelings and experiences over the past 2 weeks, using a 6-point Likert scale (0 = At no time, 1 = Some of the time, 2 = Less than half of the time, 3 = More than half of the time, 4 = Most of the time, and 5 = All of the time). We utilized a Chinese version of the WHO-5 which demonstrated strong internal consistency in the current study (Cronbach’s α > 0.9).

#### 2.2.5 Loneliness

In this study, we assessed loneliness using the UCLA Loneliness Scale-8 (ULS-8). This scale comprises eight items designed to measure the extent to which individuals experience loneliness and social isolation. Participants were asked to rate the frequency of specific thoughts and emotions related to loneliness over the past 2 weeks, using a 4-point Likert scale (0 = Never, 1 = Rarely, 2 = Sometimes, 3 = Often). We utilized a Chinese version of the ULS-8 which demonstrated acceptable internal consistency in the current study (Cronbach’s α = 0.73).

#### 2.2.6 Insomnia

In this study, we assessed life insomnia using the Insomnia Severity Index-7 (ISI-7). This questionnaire consists of seven self-report items, each addressing a specific aspect of insomnia symptoms. Participants were asked to rate the severity of each symptom over the past 2 weeks, with scores ranging from 0 to 4 (0 = No problem, 1 = Mild, 2 = Moderate, 3 = Severe, and 4 = Very severe). We utilized a Chinese version of the ISI-7 which demonstrated strong internal consistency in the current study (Cronbach’s α > 0.9).

#### 2.2.7 Physical activity

Physical activity was evaluated via frequency (31), and the questions were as follows:

“In the last month, how often did you engage in physical activity each week?” A 7-point Likert scale was used to collect answers, where 1 = never, 7 = almost every day.

#### 2.2.8 Douyin usage at bedtime

Douyin usage at bedtime was evaluated via frequency and duration of the event (32), and the questions were as follows:

Frequency: “In the last month, how often did you use Douyin at bedtime each week?” A 7-point Likert was used to collect answers, where 1 = never, 7 = almost every day.

Duration: “How long do you typically use Douyin at bedtime each day?” Answers were collected in 7 categories: 1 = less than 15 min, 2 = 15 to 30 min, 3 = 30 to 45 min, 4 = 45 to 60 min, 5 = 60 to 75 min, 6 = 75 to 90 min, and 7 = more than 90 min.

### 2.3 Statistical analysis

Given the nonnormal data, Spearman’s rank-order correlation was used to probe for general correlations between variables.

Structural equation modeling (SEM) was employed to examine the hypothesized directional paths in the conceptual frameworks. According to Bagozzi and Yi (33), our sample size of 3,392 exceeded the recommended size of twice the number of model parameters (*n* = 9).

Variance Inflation Factor (VIF) values smaller than 5.0 were considered evidence of the absence of multicollinearity (34). Based on this rule, no multicollinearity was observed among the independent variables (VIF < 3.0).

Given our sample size and the presence of multivariate non-normality, we used an asymptotically distribution-free (ADF)/weighted least squares (WLS) estimator for analysis (35, 36). The bootstrap method with 10,000 replications was used to generate corresponding standard errors and confidence intervals for all paths (37–39).

Based on the ADF/WLS estimator, the goodness of fit was assessed using the following indices (36, 40): standardized root mean square residual (SRMR) < 0.08; Tucker–Lewis index (TLI) > 0.95; goodness-of-fit index (CFI) > 0.95, and root mean square error of approximation (RMSEA) < 0.05. We did not employ the χ2 test because it is strongly affected by sample size and violation of the multivariate normality assumption (41–43).

Factor loadings for latent variables in the conceptual model >0.5 were considered acceptable. An indirect effect (i.e., a product of coefficients for the constituent links) that significantly exceeded zero was evidence of mediation (44, 45).

All statistical analyses were conducted using SPSS 26.0 and AMOS 23.0 software (SPSS Inc., Chicago, IL, United States).

## 3 Results

### 3.1 Characteristics of respondents

The final analysis included a total of 3,392 eligible respondents, with 73.53% were males and 26.47% were females (Table 1). Approximately 78.86% of the respondents fell within the age range of 18 to 30 years old. Only one respondent reported not using Douyin at night, and only 18.99% of respondents stated that they did not use Douyin at bedtime. According to the 2022 Ultimate Guide to China Social Media, as of September 2021, the largest proportion of monthly active users on Douyin falls within the age range of 25–34 years old, which aligns with the characteristics of our sample (46). However, it is important to note that this guide reports a balanced gender distribution (53% male and 47% female) (46). In contrast, our sample does not exhibit a balanced gender distribution, indicating that it may not entirely represent Chinese Douyin users.

**Table 1:** Respondent characteristics.

Variable	Category	n	Percentage
Gender	Male	n = 2,494	73.53%
	Female	n = 898	26.47%
Age (year)			
	18 ~ 30	n = 2,675	78.86%
	31 ~ 59	n = 710	20.09%
	>60	n = 7	0.01%
Frequency of physical activity			
	Never	n = 431	12.71%
	Rarely	n = 804	23.70%
	Occasionally	n = 894	26.36%
	Sometimes	n = 586	17.28%
	Often	n = 297	8.76%
	Very often	n = 111	3.27%
	Almost every day	n = 269	7.91%
Using Douyin at night			
	None	n = 1	0.00%
	Yes	n = 3,391	100.00%
Frequency of Douyin usage at bedtime			
	Never	n = 644	18.99%
	Rarely	n = 838	24.71%
	Occasionally	n = 813	23.97%
	Sometimes	n = 466	13.74%
	Often	n = 184	5.42%
	Very often	n = 70	2.06%
	Almost every day	n = 377	11.11%
Duration of Douyin usage at bedtime (minutes)			
	0 ~ 15	n = 842	24.82%
	16 ~ 30	n = 903	26.62%
	31 ~ 45	n = 878	25.88%
	46 ~ 60	n = 432	12.74%
	61 ~ 75	n = 195	5.75%
	76 ~ 90	n = 50	1.47%
	>90	n = 92	2.71%
		Mean	SD
Insomnia (ISI-7)		6.61	5.43
Life satisfaction (SWLS-5)		27.52	6.14
Well-being (WHO-5)		20.05	5.75
Depression (PHQ-9)		16.88	6.12
Anxiety (GAD-7)		6.48	4.72
Loneliness (ULS-8)		18.20	4.30

### 3.2 Correlations between variables

**Figure 2:**
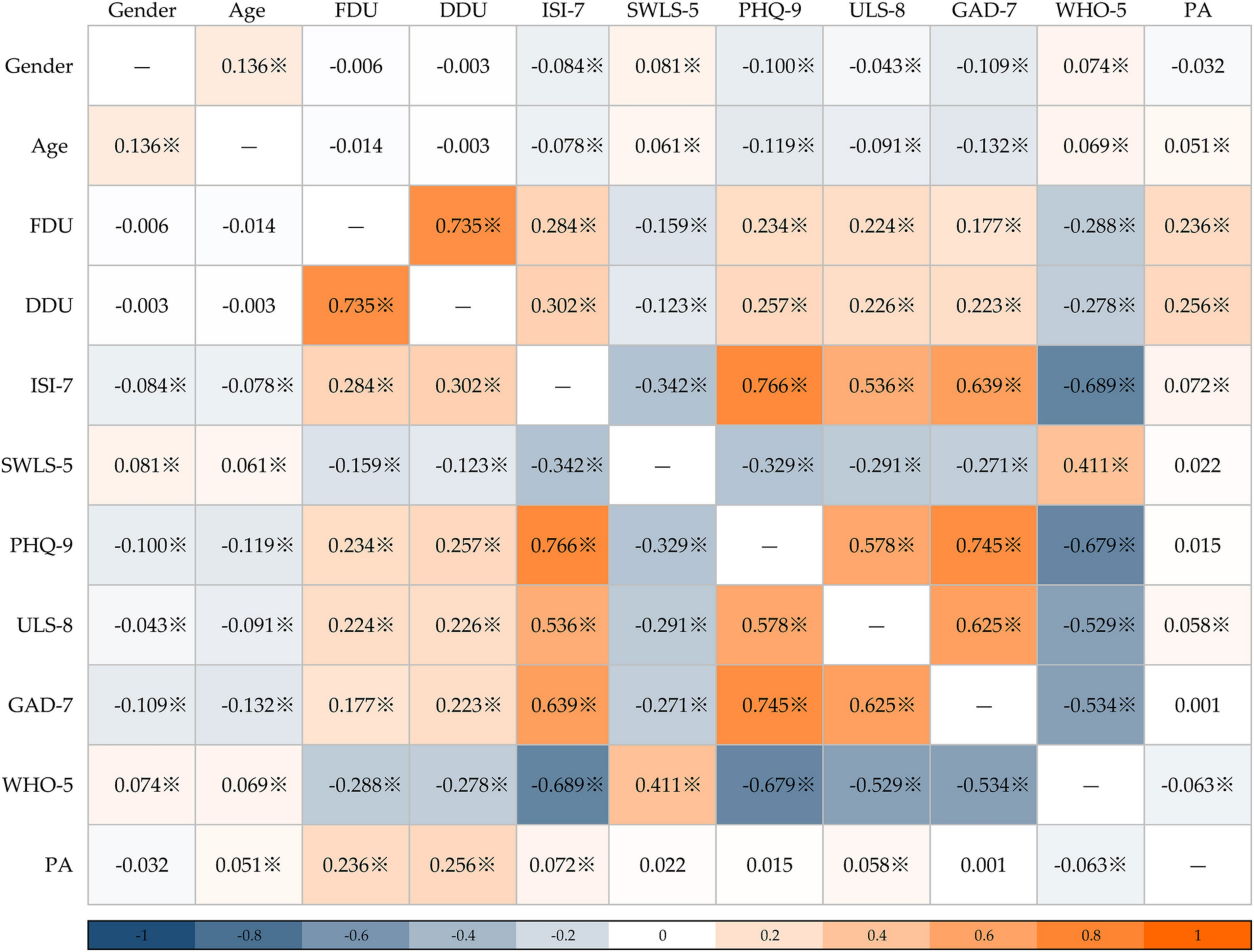
Correlation heatmap (※, *p* < 0.05; FDU, frequency of Douyin usage at bedtime; DDU; duration of Douyin usage at bedtime; PA, physical activity).

According to Figure 2, the frequency and duration of Douyin usage at bedtime were significantly correlated with insomnia, depression, anxiety, loneliness, well-being, life satisfaction, and physical activity. Moreover, insomnia was significantly correlated with gender, age, depression, anxiety, loneliness, well-being, life satisfaction, and physical activity.

### 3.3 Results of the SEM analysis

#### 3.3.1 Model modification and factor loading

**Figure 3:**
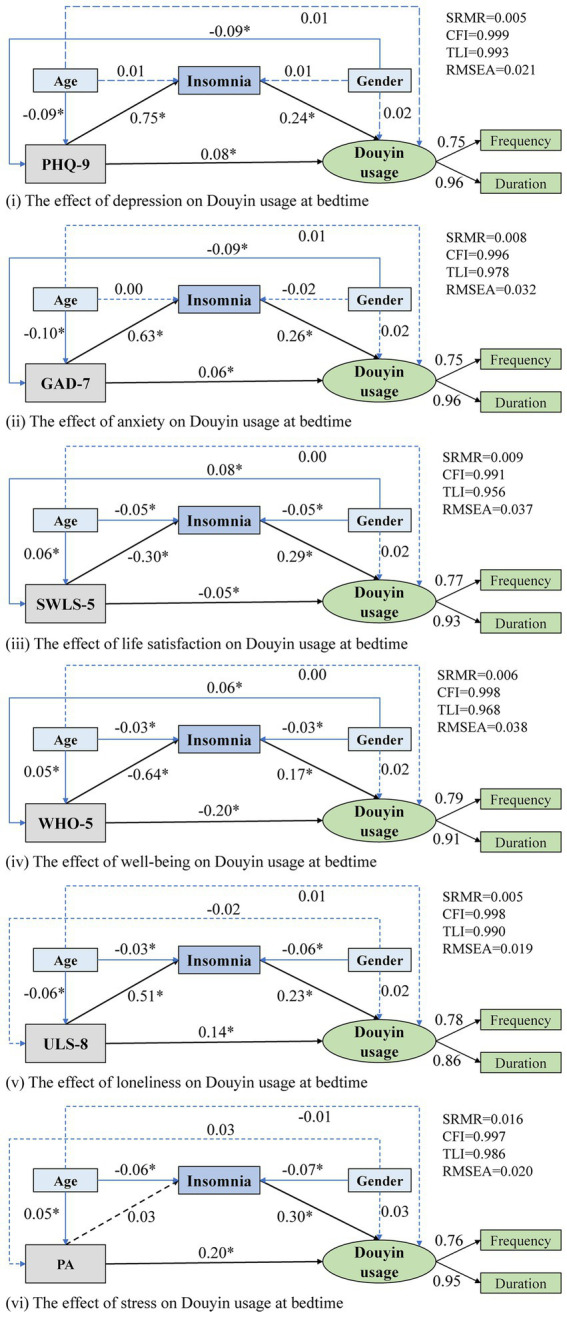
Results of path analysis and model fitting.

According to Figure 3, all six models showed good model fit (SRMR <0.08, TLI > 0.95, CFI > 0.95, and RMSEA <0.05). In addition, the factor loadings for latent variables are within an acceptable range (factor loading >0.5).

#### 3.3.2 Direct effect, indirect effect, and total effect

##### 3.3.2.1 Depression

According to Figure 3 and Table 2, depression was directly associated with increased Douyin usage at bedtime (*β* = 0.08; *p* < 0.05), and an indirect association via insomnia was also observed (*β* = 0.18; *p* < 0.05). In this model, the mediation proportion amounted to 69% of the total effect.

**Table 2:** The total and indirect effect of the SEM analysis.

Pathway	β (95% CI)	*p*
Total effect		
Depression → Douyin usage at bedtime	0.26 (0.23 to 0.30)	< 0.05
Anxiety → Douyin usage at bedtime	0.22 (0.19 to 0.26)	< 0.05
Life Satisfaction → Douyin usage at bedtime	−0.13 (−0.17 to-0.09)	< 0.05
Well-Being → Douyin usage at bedtime	−0.31 (−0.35 to 0.27)	< 0.05
Loneliness → Douyin usage at bedtime	0.26 (0.22 to 0.30)	< 0.05
Physical activity→ Douyin usage at bedtime	0.21 (0.16 to 0.25)	< 0.05
Indirect effect		
Depression → Insomnia → Douyin usage at bedtime	0.18 (0.13 to 0.23)	< 0.05
Anxiety → Insomnia → Douyin usage at bedtime	0.16 (0.13 to 0.20)	< 0.05
Life Satisfaction → Insomnia → Douyin usage at bedtime	−0.09 (−0.10 to-0.07)	< 0.05
Well-Being → Insomnia → Douyin usage at bedtime	−0.11 (−0.15 to-0.08)	< 0.05
Loneliness → Insomnia → Douyin usage at bedtime	0.12 (0.09 to 0.14)	< 0.05
Physical activity → Insomnia → Douyin usage at bedtime	0.01 (−0.00 to 0.02)	= 0.16

##### 3.3.2.2 Anxiety

According to Figure 3 and Table 2, anxiety was directly associated with increased Douyin usage at bedtime (*β* = 0.06; *p* < 0.05), and an indirect association mediated by insomnia was also observed (*β* = 0.16; *p* < 0.05). In this model, the mediation proportion amounted to 72% of the total effect.

##### 3.3.2.3 Life satisfaction

According to Figure 3 and Table 2, life satisfaction was directly associated with decreased Douyin usage at bedtime (*β* = −0.05; *p* < 0.05), and it was also indirectly associated with lower Douyin usage at bedtime via insomnia (*β* = −0.09; *p* < 0.05). In this model, the mediation proportion amounted to 69% of the total effect.

##### 3.3.2.4 Well-being

According to Figure 3 and Table 2, well-being was directly associated with decreased Douyin usage at bedtime (*β* = −0.20; *p* < 0.05), and it was also indirectly associated with lower Douyin usage at bedtime via insomnia (*β* = −0.11; *p* < 0.05). In this model, the mediation proportion amounted to 35% of the total effect.

##### 3.3.2.5 Loneliness

According to Figure 3 and Table 2, loneliness was directly associated with increased Douyin usage at bedtime (*β* = 0.14; *p* < 0.05), and it was also indirectly associated with lower Douyin usage at bedtime via insomnia (*β* = 0.12; *p* < 0.05). In this model, the mediation proportion amounted to 46% of the total effect.

##### 3.3.2.6 Physical activity

According to Figure 3 and Table 2, physical activity was directly associated with increased Douyin usage at bedtime (*β* = 0.20; *p* < 0.05), while no substantial indirect effect was observed.

## 4 Discussion

Douyin usage at bedtime is a common behavior among Douyin users. A previous study revealed that approximately 98% of Douyin users will use Douyin at night, in which approximately 86% of Douyin users occupy their bedtime using Douyin (6). In the current study, we found that this behavior remains prevalent, with approximately 82% of users reporting Douyin usage at bedtime. However, there is limited evidence regarding the reasons why people choose to use Douyin at bedtime, despite increasing interest from researchers (4). To bridge this gap, we conducted the present study to explore the potential factors and mechanisms that drive Douyin usage at bedtime. Our findings demonstrate the significance of depression, anxiety, loneliness, and physical activity as important factors contributing to Douyin usage at bedtime, while life satisfaction and well-being act as inhibitory factors that might mitigate such usage.

### 4.1 Depression and anxiety

Depression and anxiety are common mental health problems that are usually associated with problematic smartphone usage and social media usage (47, 48). In this regard, Maguire and Pellosmaa (49) discovered that individuals with more severe mental health problems tend to Douyin addiction. To further clarify the impact of mental health on domain-specific Douyin usage behaviors and the potential mechanisms involved, we conducted the current study. Our findings indicate that depression (*β* = 0.08; *p* < 0.05) and anxiety (*β* = 0.06; *p* < 0.05) may directly lead to increased Douyin usage at bedtime. Additionally, depression (*β* = 0.18; *p* < 0.05) and anxiety (*β* = 0.16; *p* < 0.05) may exert an indirect influence on Douyin usage at bedtime through the mediating role of insomnia. Notably, the indirect pathway is more prominent, accounting for approximately 69 to 72% of the total effect. A similar finding was also observed in the previous study by Zhang et al. (6), where depression and anxiety directly were associated with problematic Douyin usage at bedtime. One possible explanation is that individuals may turn to Douyin as a tool to alleviate their adverse mental health conditions and insomnia induced by these conditions, highlighting the potential positive aspects of Douyin usage. A recent case study by (50) partially supports this speculation. Their tracking survey revealed a negative relationship between social media usage at bedtime and the time to fall asleep, potentially indicating an improvement in insomnia.

It is worth noting that our findings suggest that males and young Douyin users may experience more severe depression and anxiety. This result is similar to previous research based on non-Douyin users. For instance, a study by Krokstad et al. (51) on the changing trends in mental health over the past three decades among Norwegians revealed that young individuals tend to exhibit poorer mental health. Additionally, a cross-sectional study by Kaneko and Motohashi (52) also discovered that males had worse mental health. In the context of our model, these findings imply that males and young Douyin users may exhibit increased insomnia symptoms and a greater reliance on Douyin.

### 4.2 Life satisfaction and well-being

Our findings indicate that life satisfaction (*β* = −0.05; *p* < 0.05) and well-being (*β* = −0.20; *p* < 0.05) may be directly associated with Douyin usage at bedtime, suggesting that they may act as potential protective factors against excessive Douyin use at night. Our findings are partly supported by previous studies focused on traditional social media. For example, Research by Sahin (53) and Geraee et al. (54) revealed a negative correlation between life satisfaction and social media addiction. Brooks (55) also observed a similar relationship between well-being and social media usage. These findings imply that excessive social media usage, including Douyin usage at bedtime, could potentially benefit from an improved overall state of physical and mental health.

As mentioned above, many studies have investigated the relationship between social media usage and life satisfaction, as well as well-being (53–55). Meanwhile, the connection between insomnia and life satisfaction, as well as well-being has also been uncovered (20, 56). However, less is known about the mediating role of insomnia between these factors. In response, we conducted the current study to address this gap. Our findings suggest that life satisfaction (*β* = −0.09; *p* < 0.05) and well-being (*β* = −0.11; *p* < 0.05) may improve Douyin usage at bedtime via alleviating insomnia. It is worth noting that our study found that life satisfaction was primarily linked to Douyin usage through an indirect pathway (approximately 69% mediation proportion), while well-being was primarily linked to Douyin usage through a direct pathway (approximately 35% mediation proportion). The reasons for this discrepancy remain unclear. Furthermore, our results indicate that females and older Douyin users tend to have higher levels of life satisfaction and well-being, implying better overall health status and reduced reliance on Douyin.

### 4.3 Loneliness

In the past decade, loneliness has been considered one of the major factors contributing to internet addiction and problematic social media usage (13, 57). In this aspect, several studies have explored the association between loneliness and Douyin usage, finding a positive relationship between them (58, 59). However, there is limited knowledge about the specific mechanisms through which loneliness influences Douyin usage. To bridge this gap, our study investigated the effect of loneliness on Douyin usage at bedtime and the potential mediating role of insomnia. Our study found that loneliness (*β* = 0.14; *p* < 0.05) may directly contribute to increased Douyin usage at bedtime. Moreover, insomnia (*β* = 0.12; *p* < 0.05) appears to play a significant mediating role in this relationship. Interestingly, in this mediation model, the direct and indirect effects are almost equal, with the mediation accounting for approximately 46% of the total effect. In conclusion, both loneliness and loneliness-induced insomnia may drive Douyin users to use Douyin at bedtime.

Furthermore, in line with previous research involving the general public, males and young individuals might experience higher levels of loneliness (60, 61). Our findings indicate that male and young Douyin users may have higher levels of loneliness than their counterparts, which implies that they may have stronger motivations for using Douyin at bedtime.

### 4.4 Physical activity

Regular physical activity has long been regarded as an effective strategy for improving both physical and mental health (62, 63). Some researchers also believe that regular exercise has a positive impact on alleviating internet and social media addiction (64, 65). However, our findings indicate that physical activity (*β* = 0.20; *p* < 0.05) was directly associated with increased Douyin usage at bedtime. Moreover, no significant indirect effect was observed in our model (*p* < 0.05), which means physical activity may not impact Douyin usage at bedtime via insomnia. The reasons behind this finding may be related to the specific population we included. In the current study, we included active Douyin users who have used Douyin at least 1 h per day in the past month. In this context, a recent study found that physical activity was more effective in improving social media usage among individuals with low levels of social media addiction (66). However, among those with high levels of social media addiction, physical activity may act as a catalyst for increasing social media usage (66). To better understand the role of physical activity in improving Douyin usage at bedtime, we recommend that future research conducts a comparative or stratified analysis based on Douyin addiction levels. This could provide valuable insights into the complex relationship between physical activity and Douyin usage.

### 4.5 Limitations

Some limitations should be noted when interpreting our findings. First, our study had an unbalanced gender distribution, with 78.86% being male. This may not accurately reflect Chinese Douyin users. Therefore, our findings may not be entirely applicable to Chinese Douyin users. Second, our study only included Douyin users who used Douyin for at least 1 h per day in the past month. Therefore, our findings cannot be generalized to the general public. Third, our study used a chain referral method to obtain data, which may result in self-selection bias. This is because participants may be influenced by those who have already participated in the study. Fourth, we only used self-reported measures due to our limited experimental conditions, so reporting bias must have existed. Some variables, such as the duration of Douyin usage at bedtime, can be re-investigated with objective measures. Finally, our study used a cross-sectional design, which limits our ability to establish causal relationships between variables. This is an inherent limitation of analyzing such data. To re-examine the causality in each path, longitudinal trials or controlled trials are warranted.

## 5 Conclusion

This study aimed to investigate the factors driving Douyin usage at bedtime and explore potential mechanisms. Our results revealed that high levels of depression, anxiety, loneliness, and low levels of life satisfaction and happiness may drive people to use Douyin at bedtime. Insomnia appears to be a significant mediator between the factors of interest and Douyin usage at bedtime. One possible explanation is that Douyin users might turn to Douyin as a tool to alleviate their adverse physical and mental health conditions. Additionally, we found that engaging in physical activity did not improve Douyin usage; in fact, it might lead to increased usage. The specific reasons for this require further exploration. In summary, our findings offer a unique perspective on why Douyin users are drawn to using Douyin at bedtime.

## Data availability statement

The raw data supporting the conclusions of this article will be made available by the authors, without undue reservation.

## Ethics statement

The studies involving humans were approved by the Ethics Review Board of Southwest University. The studies were conducted in accordance with the local legislation and institutional requirements. The participants provided their written informed consent to participate in this study.

## Author contributions

SS: Writing – original draft, Writing – review & editing. HL: Writing – review & editing. SF: Writing – review & editing. RP: Writing – review & editing. XZ: Writing – original draft, Writing – review & editing. HL: Writing – review & editing, Investigation. MZ: Writing – review & editing. MY: Writing – review & editing.
